# Liposomal Nasal Spray versus Guideline-Recommended Steroid Nasal Spray in Patients with Chronic Rhinosinusitis: A Comparison of Tolerability and Quality of Life

**DOI:** 10.1155/2014/146280

**Published:** 2014-05-22

**Authors:** Anna Eitenmüller, Lisa Piano, Myriam Böhm, Kija Shah-Hosseini, Andreas Glowania, Oliver Pfaar, Ralph Mösges, Ludger Klimek

**Affiliations:** ^1^Institute of Medical Statistics, Informatics and Epidemiology, University of Cologne, 50924 Cologne, Germany; ^2^Ear, Nose and Throat Department, General Hospital Hietzing, 1130 Vienna, Austria; ^3^Center for Rhinology and Allergology, 65183 Wiesbaden, Germany

## Abstract

*Objective*. To investigate the tolerability and impact on quality of life of liposomal nasal spray compared to guideline-recommended steroid-based therapy in patients with chronic rhinosinusitis. Symptom reduction and use of antisymptomatic medication were also examined. *Methods.* In this monocenter, prospective, controlled, open, and noninterventional study, 60 patients with chronic rhinosinusitis were treated with liposomal nasal spray and 30 patients received steroid-based therapy. The study comprised five visits occurring at intervals of two to four weeks. Efficacy was determined according to the sinusitis symptom score documented daily. The polyp score was recorded at the initial and final visits. Tolerability was determined through the Nasal Spray Evaluation Questionnaire, and quality of life was ascertained with the SNOT-20 Score. *Results*. Both treatments achieved a significant reduction of sinusitis symptoms (*P* < 0.05) and also rhinoscopic improvement (*P* < 0.05). The majority of patients assessed the treatments as “good” or “very good,” and the quality of life improved significantly (*P* < 0.05). There was no significant difference in symptom reduction, QoL, and endoscopic exams between both treatments. *Conclusion*. The treatment of chronic rhinosinusitis with liposomal nasal spray results in a similar, significant reduction of symptoms and significant improvement in quality of life as guideline-recommended treatment and is therefore a comparable alternative.

## 1. Introduction

With a lifetime prevalence of about 5%, chronic rhinosinusitis (CRS) is one of the most frequently occurring chronic disorders worldwide [1, 2]. The German, European, and US-AWMF guidelines recommend as treatment the topical application of glucocorticoids since they represent an important treatment principle in addition to antibiotic treatment in conservative therapy [2, 3]. Nasal irrigation or sprays with hypertonic buffered solutions can also provide symptom relief in CRS disorders and are therefore recommended by guidelines. These sprays improve mucociliary clearance by liquefying nasal secretion and have been observed to have vasoconstrictive and decongestant effects [4].

Treatment alternatives should be pointed out to patients who have a critical view of guideline-recommended steroid-based therapy. One such alternative therapy concept is the nasal application of (phospholipid) liposomes. Several studies have already demonstrated the efficacy of this nonpharmacological mechanism of action in allergic rhinitis [5, 6]. Three precursor studies which investigated the application of a liposomal nasal spray in patients with seasonal allergic rhinitis showed significant symptom improvement and good tolerability of the liposomal nasal spray, also in comparison to guideline combination therapy with glucocorticoids and antihistamines [7–9].

Because the incidence of allergic rhinitis (AR) in adults with CRS is 40%–80%, liposome therapy therefore represents an interesting, steroid-free treatment alternative [10]. Since two-thirds of patients with AR alone have steroid phobia, the probability is high that the fear of medication containing cortisone also exists among patients with CRS [11]. The liposomes, produced from phosphatidylcholine, stabilize the surfactant film and prevent the moisture film lining the airways from tearing. A liposomal nasal spray (LN) therefore represents an entirely drug-free treatment concept [12]. The present study investigates symptom reduction after the application of a LN in patients with CRS. Tolerability and the impact on quality of life were also determined.

The study was carried out in compliance with the requirements for noninterventional studies [13]. Since both products can be purchased without a prescription, it was not necessary to seek approval from an ethics committee. Nevertheless, a consultation with the competent ethics committee with respect to professional regulations took place before the study commenced.

## 2. Methods

### 2.1. Study Design

This investigation was a monocenter, prospective, controlled, open, noninterventional study (NIS). Sixty patients with CRS symptoms were treated with LN, and 30 patients received guideline-recommended therapy with a steroid nasal spray. Patients were first offered the guideline-recommended therapy. Those patients having reservations towards pharmacological therapy were alternatively offered treatment with a liposomal nasal spray. No prior wash-out period was required. Patients 18 years or older were included who due to their disorder had already been undergoing treatment at the study center.

The NIS took place from 15 March 2011 to 31 January 2013 and consisted of five visits at intervals of two to four weeks within a total treatment period of three months. Efficacy was determined on the basis of the sinusitis symptom score (SSS), which was documented daily by the patients themselves in a patient diary; furthermore, the investigator recorded the SSS at every visit. For monitoring purposes, the investigator also determined the polyp score (PS) based on the size of polyps at the first and last visits [14]. The nasal spray sensory scale was used at the first and last visits to assess tolerability [15]. Quality of life (QoL) was determined at every visit by means of the SNOT-20 score [16].

### 2.2. Medication

The liposomal nasal spray* LipoNasal Pflege* (LN), manufactured by Optima Pharmazeutische GmbH, Moosburg/Wang, Germany, was applied in this study. The liposomes contained in this product consist of highly purified soy lecithin, which is composed of 94% phosphatidylcholine and a small proportion of other phospholipids. Other components of the nasal spray are sodium chloride, ethanol, dexpanthenol, vitamin A palmitate, tocopherol, and water for injection. Treatment was carried out according to leaflet instructions, with the spray being applied an average of 2-3 sprays per nostril daily.

The comparative treatment used in this study was* Livocab direkt mit Beclometason 0.05% *(LB), a corticoid having an anti-inflammatory effect for nasal application, manufactured by Orion Corporation Orion Pharma, Finland. This preparation is primarily applied short-term for seasonal allergic rhinitis. Its active ingredient is beclomethasone, which in this product is available as beclomethasone dipropionate. Beclomethasone is a synthetic glucocorticoid with vasoconstrictive, immunosuppressive, antiallergic, and anti-inflammatory properties that is used to treat asthma, allergic rhinitis, and sinusitis. Beclomethasone dipropionate is used as a prodrug and is subject to a first-pass effect in the liver, thereby limiting toxicity and systemic bioavailability.

Other components of the product are benzalkonium chloride, polysorbate 80, D-glucose, microcrystalline cellulose, carmellose sodium, purified water, sodium hydroxide, and hydrochloric acid for pH regulation.

One milliliter of nasal spray contains 0.555 mg (approx. 0.05%) beclomethasone dipropionate as the active ingredient.

One spray application (approx. 0.09 mL) contains 0.05 mg beclomethasone dipropionate.

The recommended dose for patients aged 12 years or older is 2 sprays per nostril and application. The maximum daily dosage is 4 sprays per nostril.

### 2.3. Study Protocol

On Day 1 of treatment (Visit 1), the investigator documented the detailed medical history and the SSS as well as the PS and conducted a regular rhinoscopy. Videoendoscopy and/or a smell test were optional.

Patients documented the number of sprays applied per nostril daily. They also specified when an onset of action occurred after the first-time application of the nasal spray (<5 min, 5–10 min, 10–30 min, 30–60 min, 1-2 h, 2–4 h, 4–8 h, >8 h, no onset of action).

Efficacy was recorded by means of the SSS, which was chosen based on the EPOS Paper [17]. The present study, however, implemented a slightly adapted version of the score to enable a direct comparison with another study that investigated steroid treatment of CRS [18]. The score was recorded at every visit by the physician as well as daily by the patient in a patient diary. The five main symptoms of rhinosinusitis (rhinorrhea, nasal obstruction, headache, facial pain, and postnasal drip) were evaluated on the basis of an ordinal scale from 0 to 3 (0 = no, 1 = mild, 2 = moderate, and 3 = severe), and the individual values were added together to obtain a sum score. Furthermore, a rhinoscopic examination was conducted at every visit to ensure an additional objective assessment of efficacy. In the process, the symptoms “edema,” “secretion,” and “redness” were evaluated on a 3-point scale (0 = no, 1 = mild, and 2 = severe), and the rhinoscopy score (RS) was calculated thereafter from the data obtained.

In addition, polyps were measured via endoscopy at the first and last visits to monitor polyp size. The PS was calculated from these data based on a 4-point scale (0 = no polyps, 1 = small polyps, 2 = medium-sized polyps, and 3 = large polyps) [14].

The tolerability of the nasal spray was determined by means of the Nasal Spray Sensory Scale [15]. Patients answered 14 questions pertaining to sensory parameters on a visual analog scale (0 = poor evaluation and 100 = good evaluation) immediately after applying the nasal spray as well as two minutes thereafter.

Quality of life was recorded using a validated questionnaire, the “Sino-Nasal Outcome Test German Adapted Version” (SNOT-20 GAV), which patients completed at every visit [19, 20]. This form consists of 20 individual questions relating to symptoms as well as social and emotional consequences, which the patient was able to assess on a 6-point scale (0 = no problem, 1 = very minor problem, 2 = small problem, 3 = moderate problem, 4 = severe problem, and 5 = it cannot get any worse). From these 20 individual questions, the patients were also able to choose the five items most important to them. In addition, the patients assessed their subjective condition daily on a visual analog scale (0 = very poor and 100 = very good).

At Visit 5, a final evaluation was made during which the investigator assessed the medication applied in terms of effect and tolerability. Patients were also able to evaluate tolerability and efficacy of the nasal spray at the end of the treatment period with a final diary entry.

### 2.4. Statistics

The program SPSS 21 for Windows was used to conduct the statistical analysis. To reduce any input errors, double data entry was carried out. Unreported values were treated as “missing values.”

First, all data were analyzed descriptively and tested for normal distribution using the Kolmogorov-Smirnov test. In addition, the mean values of the variables from Visits 1 and 5 were compared with the aid of the *t*-test for paired samples. The level of significance was set at *α* = 0.05.

## 3. Results

### 3.1. Homogeneity of Treatment Groups

Overall, 35 women and 25 men aged 18 to 77 years (mean age = 42 years) were included in the LN group, and 16 women and 14 men aged 22 to 74 years (mean age = 46 years) were in the comparison group. The statistical analysis and the comparison of the demographic data showed no relevant differences between the groups at the beginning of treatment (Table 1). The analysis of the symptom scores for the previous year revealed that most patients suffered from rhinoconjunctivitis complaints (LN = 55.2% and comparison group = 51.7%) and also from asthma, rhinoconjunctivitis, and conjunctivitis. Allergies were also frequent (LN = 54.2% and comparison group = 51.7%) (Table 2). Overall, 22% in the LN group and 30% in the comparison group suffered from polyps.

### 3.2. Onset of Action

In the LN group, the onset of action on Day 1 occurred within 30 minutes in 47.8% of the patients, with 39.1% not noticing any onset of action at all.

The onset of action on Day 1 in the beclomethasone group took place within 30 minutes in 20% of the patients, with 48% noticing no onset of action whatsoever.

### 3.3. Efficacy

**Figure 1:**
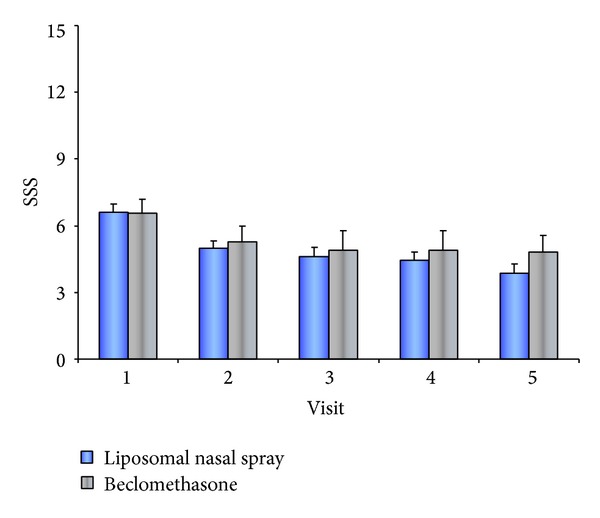
Sinusitis symptom score over the course of five visits in both groups.

The liposomal nasal spray and the steroid alternative were both able to improve sinusitis symptoms significantly, with rhinoscopy findings also demonstrating distinct improvement. The sinusitis symptom score in the LN group, for instance, declined from a baseline score from 6.61 (±2.668) to 3.88 (±3.674) and in the comparison group from 6.57 (±3.012) to 4.83 (±3.601) (see Table 3 and Figure 1). The sum score of the rhinoscopic evaluation also decreased in the LN group from 3.78 (±1.368) to 1.85 (±1.477) and in the steroid group from 4.26 (±1.096) to 2.30 (±1.222) (see Table 3). The analysis of the polyp scores showed no relevant change with respect to polyp size.

No relevant differences with regard to symptom reduction could be determined in the statistical analysis of the patient diaries.

Overall, the morning SSS, which consisted of the diary items “runny nose,” “itching,” “sneezing,” “postnasal drip,” “facial pain,” “headache,” and “nasal obstruction,” was 4.06 in the LN group and 4.01 in the steroid group out of 15 possible points. Over the three-month observation period, the patients' daily documented sinusitis sum scores in both groups decreased from approximately 4 at baseline to 2.16 on Day 86. The difference between both groups was not significant.

At the final evaluation, the majority of the patients in both groups rated efficacy “good” or even “very good” (Table 7).

### 3.4. Tolerability and Safety

In the immediate evaluation of the Nasal Spray Sensory Scale, the steroid nasal spray group achieved somewhat better results than the LN group (*P* = 0.178), but ultimately there was no significant difference between both groups at V5 (*P* = 0.564) (Table 4).

In the evaluation after two minutes, both groups showed higher values, which means that the application was perceived as more pleasant. The value for the LN group was 80.4 at Visit 1 and 78.8 at Visit 5. In the group receiving beclomethasone, the value was 85.1 at Visit 1 and 78.3 at Visit 5. No significant difference existed between both nasal sprays comparing its tolerability immediately (*P* = 0.594) or after 2 minutes (*P* = 0.815), neither at V1 nor at V5 (Table 4).

In the final assessment of tolerability, the majority of evaluable patients in both groups rated both treatments “good” or even “very good” (Table 7).

Overall, both treatment modalities were tolerated well; no significant difference between both groups was observed (*P* = 0.306).

### 3.5. Dropouts and Adverse Events

Altogether, 20 patients from the LN group and seven patients from the cortisone group dropped out of the study. In most cases, the reasons for discontinuation remained unknown; only one patient from the LN group and two patients from the cortisone group dropped out of the study because of an adverse event (AE). AEs occurred in a total of 23 patients, 10 events of which were reported in the LN group and 13 in the group receiving the steroid nasal spray.

One patient from the LN group and five patients from the beclomethasone group also experienced a second AE (Table 5).

None of these incidents were documented by the study investigators as serious adverse events in the serious adverse event (SAE) form. An association with the study drug could not be ruled out for five AEs in the beclomethasone and eight AEs in the LN group.

### 3.6. Quality of Life

The application of both preparations resulted in a significant improvement in quality of life as early as V2 (*P* ≤ 0.05). The treatments themselves did not differ from each other significantly (*P* ≥ 0.05).

**Figure 2:**
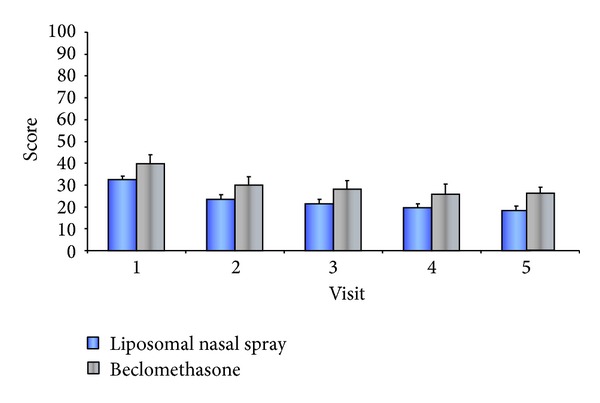
Course of the SNOT total score V1 to V5.

In the LN group, therapy caused a drop in the total sum score of the SNOT-20 Quality of Life Scale from 32.57 ± 10.786 to 18.43 ± 13.372; the score decreased in the comparison group from 39.91 ± 19.776 to 26 ± 22.076 (see Table 3 and Figure 2). When dividing the SNOT-20 Quality of Life Scale into “primary nasal symptoms,” “secondary rhinogenic symptoms,” and “general quality of life,” significant improvements could also be observed within these subareas (*P* ≤ 0.05) (Table 6). No significant difference existed between the groups.

Besides the evaluation of the SNOT-20, patients recorded their subjectively perceived condition daily in a diary as a further parameter for determining quality of life.

**Figure 3:**
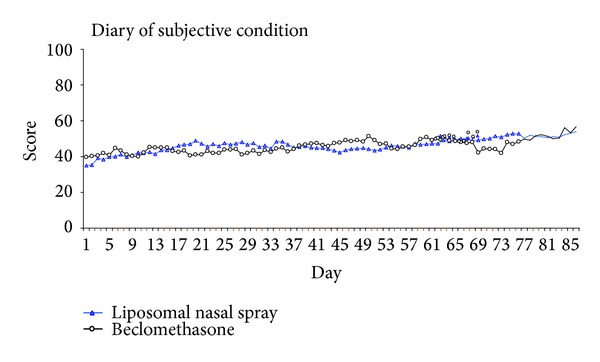
Diary assessment of the subjective condition of patients.

Figure 3 shows the three-month course of the patients' mean subjective condition over the treatment period.

## 4. Discussion

Besides antibiotics, topical treatment with corticosteroids is the guideline-recommended treatment of choice for symptomatic CRS [2], although so-called “cortisone phobia” represents an increasing problem. This circumstance often results in lacking patient compliance and the change to an alternative steroid-free medication [21]. As demonstrated in previous studies on allergic rhinitis and rhinoconjunctivitis, therapy with a liposomal nasal spray is an equally effective and tolerable treatment alternative [7–9]. In terms of efficacy, it is assumed that the phospholipids supplemented via nasal spray stabilize or restore the impaired “nasal surfactant,” thereby maintaining the natural moisture film protecting and moisturizing the nasal mucosa and as a basis of mucociliary clearance [12]. The present study was able to illustrate that the application of liposomes also represents a promising treatment option for CRS. Since CRS is often accompanied by allergic rhinitis, a possible explanatory approach may also lie in the wound-healing and anti-inflammatory properties of liposomes [22]. The barrier function of the nasal mucus layer is impaired due to the inflamed mucosa. Liposomes primarily consist of phosphatidylcholine, which in terms of quantity make up the largest proportion of nasal surfactant and are thus able to stabilize the liquid film that moisturizes and protects the mucus membrane [12].

Since symptom scores can always be subjectively influenced [23] and to obtain the most objective evaluation of efficacy as possible, we chose a combination of investigator evaluation and patient evaluation. The symptoms for the SSS, which patients entered in their diaries and the physician filled out at the visits, were selected based on the EPOS Paper 2007 [17]; for better comparability with a steroid, however, the study implemented a slightly adapted score [18]. Ultimately, the items “nasal secretion,” “postnasal drip,” “facial pain,” “headache,” and “nasal obstruction” were used. Application of the liposomal nasal spray led to a significant improvement in the SSS of 2.7 from V1 to V5, corresponding to improvement by 41.4%; improvement in the comparison group was 26.5%. The sum score of the rhinoscopic evaluation also decreased significantly in the LN group from 3.78 to 1.85 (51% improvement) and in the steroid group from 4.26 to 2.3 (46% improvement). In a precursor study on allergic rhinoconjunctivitis, the application of the liposomal nasal spray resulted in nasal symptom relief of 33.2% and global improvement of 41.4%, which support the results of the present study [9].

Overall, tolerability of the liposomal nasal spray was assessed positively; 50% of the valid percentages rated tolerability “good,” 39.4% “very good,” 7.9% “satisfactory,” and only 2.6% evaluated tolerability “poor.” Some patients commented on the smell of the liposomal spray. Since it was decided to deliberately forgo the addition of artificial aromas in the product to avoid possible allergic reactions or intolerances, the natural scent of lecithin (phospholipids) is perceptible.

### 4.1. Quality of Life

A study by Rudmik and Smith showed that CRS leads to a significant loss of quality of life, among other things due to symptoms such as sleeplessness, headache, and facial pain, and also emotional consequences such as sadness and a sense of shame [24]. In this study, a significant decrease resulted in the total sum score of the SNOT-20 Quality of Life Scale and in the subareas “primary nasal symptoms” and “secondary rhinogenic symptoms” as well as in the area “general quality of life.” Thus, significant improvement in quality of life could be verified through the use of a nonpharmacological product in CRS. Both preparations, however, do not differ significantly.

## 5. Conclusion

All in all, both of the applied treatments led to significant improvement in the patients' condition, with no significant differences resulting between both study medications for the most part.

The values calculated in this study show that liposomal nasal spray is an effective treatment alternative for patients with CRS. Its application resulted in significant symptom reduction and improved quality of life. Furthermore, the majority of patients assessed its tolerability very positively. Liposomal nasal spray is therefore a suitable steroid-free method for treating CRS, particularly for patients who take a somewhat critical view of guideline-recommended therapy with cortisone.

## Figures and Tables

**Table 1:** Demographic data.

	Female		Male		Valid		Missing	Total
	*n*	%	*n*	%	*n*	%	*n*	*n*
Liposomal nasal spray	*35 *	58.3	*25 *	41.7	*60 *	100	*0 *	*60 *
Beclomethasone	*16 *	53.3	*14 *	46.7	*30 *	100	*0 *	*30 *

**Table 2:** Distribution of allergies.

	Trees		Weeds		Grasses		Mites		Mold	
	*n*	%	*n*	%	*n*	%	*n*	%	*n*	%
Liposomal nasal spray										
Patients with allergy	*14 *	24.1	*2 *	3.4	*16 *	27.6	*8 *	13.8	*3 *	5.2
Patients without allergy	*44 *	75.9	*56 *	96.6	*42 *	72.4	*50 *	86.2	*55 *	94.8
Valid	*58 *	100	*58 *	100	*58 *	100	*58 *	100	*58 *	100
Beclomethasone										
Patients with allergy	*8 *	26.7	*1 *	3.3	*6 *	20	*8 *	26.7	*3 *	10
Patients without allergy	*22 *	73.3	*29 *	96.7	*24 *	80	*22 *	73.3	*27 *	90
Valid	*30 *	100	*30 *	100	*30 *	100	*30 *	100	*30 *	100

**Table 3:** Sinusitis sum score (SSS), rhinoscopy sum score (RS), and SNOT-20 total score.

	V1	V2	V3	V4	V5	Improvement V1–V5
Liposomal nasal spray						
SSS						
MV	**6.61**	**5.00**	**4.63**	**4.45**	**3.88**	**2.73**
SD	*2,668 *	*2.327 *	*2.797 *	*2.615 *	*2.674 *	*2.849 *
RS						
MV	**3.78**	**2.50**	**2.28**	**1.60**	**1.85**	**1.93**
SD	*1.368 *	*1.177 *	*1.724 *	*1.676 *	*1.477 *	1.639
SNOT						
MV	**32.57**	**23.64**	**21.51**	**19.57**	**18.43**	**14,14**
SD	*10.786 *	*12.694 *	*13.204 *	*13.590 *	*13.372 *	*12,731 *
Beclomethasone						
SSS						
MV	**6.57**	**5.26**	**4.91**	**4.91**	**4.83**	**1.74**
SD	*3.012 *	*3.441 *	*4.231 *	*4.100 *	*3.601 *	*3.151 *
RS						
MV	**4.26**	**3.04**	**2.65**	**2.87**	**2.30**	**1.96**
SD	*1.096 *	*1.261 *	*1.112 *	*1.604 *	*1.222 *	*1.147 *
SNOT						
MV	**39.91**	**30.04**	**28.04**	**25.83**	**26.00**	**13.91**
SD	*19.776 *	*18.165 *	*19.427 *	*21.777 *	*22.076 *	*19.246 *

**Table 4:** Sensory evaluation immediately and two minutes after application.

	Immediately after application		Two minutes after application	
	V1	V5	V1	V5
Liposomal nasal spray				
MV	**75.25**	**72.42**	**80.39**	**78.81**
SD	*13.933 *	*14.826 *	*16.146 *	*15.686 *
Beclomethasone				
MV	**80.34**	**73.82**	**85.13**	**78.35**
SD	*13.549 *	*16.714 *	*11.2686 *	*17.975 *

**Table 5:** Adverse events.

	(1) Adverse event	(2) Adverse event
Beclomethasone	Acute sinusitis	Acute rhinitis
	Minor hemorrhoid bleeding	Acute viral rhinopharyngitis
	Cephalgia	Acute sinobronchitis
	Acute bacterial sinusitis	Acute bacterial sinusitis
	Rhinitis, cough	
	Acute viral rhinopharyngitis	
	Viral upper respiratory tract infection	
	Acute viral infection	
	Acute exacerbation of CRS	
	Gastroenteritis	Tonsillitis
	Acute viral rhinopharyngitis with rhinosinusitis	
	Bronchitis	
	Dysesthesia of nasal mucosa and facial pain	

Liposomal nasal spray	Recurrence of chronic lymphatic leukemia	
	Acute exacerbation of chronic rhinosinusitis	
	Acute bronchitis	
	Cephalgia	Fatigue
	Rhinitis	
	Infection of paranasal sinuses	
	Capsulitis DII right hand	
	Arthrosis of both hip joints	
	Acute exacerbation of chronic pansinusitis	
	Acute sinusitis	

**Table 6:** SNOT-20 subscales:primary nasal symptoms, secondary rhinogenic symptoms, and general quality of life.

	V1	V2	V3	V4	V5	Improvement V1–V5
Liposomal nasal spray						
Primary nasal symptoms						
MV	**40.24**	**31.02**	**28.57**	**26.20**	**24.90**	**15.34**
SD	*14.976 *	*13.204 *	*17.531 *	*18.295 *	*18.042 *	*−3.066 *
Secondary rhinogenic symptoms						
MV	**32.52**	**20.68**	**21.97**	**19.52**	**18.44**	**14.08**
SD	*17.220 *	*15.811 *	*14.528 *	*14.337 *	*14.563 *	2.657
General quality of life						
MV	**29.22**	**21.42**	**18.39**	**16.78**	**15.70**	**13.52**
SD	*16.726 *	*17.451 *	*16.341 *	*16.217 *	*15.634 *	*1.092 *
Beclomethasone						
Primary nasal symptoms						
MV	**45.00**	**33.33**	**31.33**	**28.33**	**28.50**	**16.5**
SD	*3.012 *	*3.441 *	*4.231 *	*4.100 *	*3.601 *	*−0.589 *
Secondary rhinogenic symptoms						
MV	**35.00**	**28.89**	**27.64**	**25.14**	**24.86**	**10.14**
SD	*19.009 *	*17.629 *	*20.489 *	*20.990 *	*21.714 *	*−2.731 *
General quality of life						
MV	**40.10**	**29.18**	**26.57**	**25.51**	**25.99**	**14.11**
SD	*24.255 *	*21.496 *	*22.267 *	*25.662 *	*25.806 *	*−1.551 *

**Table 7:** Patients' final evaluation of efficacy and tolerability.

	Efficacy	Tolerability
Liposomal nasal spray		
Very good	17.8%	39.4%
Good	53.8%	50%
Satisfactory	17.9%	7.9%
Poor	10.3%	2.6%
Beclomethasone		
Very good	17.4%	26.1%
Good	47.8%	60.9%
Satisfactory	13%	4.3%
Poor	21.7%	8.7%

## Abbreviations

AE: Adverse event
AR: Allergic rhinitis
CRS: Chronic rhinosinusitis
LN: Liposomal nasal spray
LB: Livocab beclomethasone
NIS: Non-interventional study
PS: Polyp score
QoL: Quality of life
RS: Rhinoscopy score
SAE: Serious adverse event
SSS: Sinusitis symptom score.
